# Construction of transcriptome atlas of white yak hair follicle during anagen and catagen using single-cell RNA sequencing

**DOI:** 10.1186/s12864-022-09003-8

**Published:** 2022-12-08

**Authors:** Qingbo Zheng, Na Ye, Pengjia Bao, Xiaolan Zhang, Fubin Wang, Lanhua Ma, Min Chu, Xian Guo, Chunnian Liang, Heping Pan, Ping Yan

**Affiliations:** 1 Key Laboratory of Yak Breeding Engineering of Gansu Province, Lanzhou Institute of Husbandry and Pharmaceutical Sciences, Chinese Academy of Agricultural Sciences, Lanzhou, 730050 China; 2 Key Laboratory of Animal Genetics and Breeding On Tibetan Plateau, Ministry of Agriculture and Rural Affairs, Lanzhou Institute of Husbandry and Pharmaceutical Sciences, Chinese Academy of Agricultural Sciences, Lanzhou, 730050 China; 3 Life Science and Engineering College, Northwest Minzu University, Lanzhou, 730030 China

**Keywords:** scRNA-seq, Yak, Hair follicle, Anagen, Catagen

## Abstract

**Background:**

As the direct organ of villus, hair follicles have obvious seasonal cycles. The hair follicle cycle is orchestrated by multiple cell types that together direct cell renewal and differentiation. But the regulation property of hair follicle cells from anagen to catagen in yak is still unknown.

**Results:**

In this study, single-cell RNA sequencing was performed on 24,124 single cells of the scapular skin from white yak. Based on tSNE cluster analysis, the cell types of IFE-DC, epidermal cell lines, fibroblasts, keratinocytes, IRS, DS, INFU, and other cells in yak hair follicles during anagen and catagen were successfully identified, and the gene expression profiles were described. The GO enrichment analysis indicated the different cells characteristic genes to be mainly enriched in the epidermal development, epithelial cell differentiation and wound healing pathways. The pseudotime trajectory analysis described the differentiation trajectory of the epidermal lineage and dermal lineage of the hair follicle during anagen and catagen. Moreover, the dynamic changes of the genes like *LHX2, KRT25,* and *KRT71* were found to be highly expressed in HS and IRS, but not in the IFE-DC, INFU, and keratinocyte during differentiation.

**Conclusions:**

Our results analyzed the time-varying process of gene expression in the dermal cell lineage and epidermal cell lineage of hair follicles during anagen and catagen during fate differentiation was expounded at the single cell level, revealing the law of fate specialization of different types of cells. In addition, based on the enrichment analysis, the transcriptional regulatory factors involved in the different cell fates were also revealed. These results will help to enhance our understanding of yak hair follicle cycle and promote the development and utilization of yak villus.

## Background

The Tianzhu white yak is a unique local breed of yak in the Tianzhu Tibetan Autonomous County, Gansu Province. It is well-known for its pure white coat and reputed as the reputation of “plateau white pearl” and “Qilian snow peony” [1]. The yak villus is a special kind of high-quality wool spinning raw material which is soft, warm, and breathable. Yak is the vital means of production and living for the Tibetan people, and the development of secondary hair follicles in yaks shows seasonal periodic changes [2]. Seasonal changes stimulate hair growth and shedding. The dense hair is very important for the survival of yaks in winter. After entering the winter, yaks grow villi to resist the cold winter of the plateau. In the spring and summer, yaks lose their hair to increase the dissipation of heat. Hair follicles are an important accessory renewable organ of the skin, controlling hair growth. During the hair follicle formation, a series of signaling molecules shuttle between the dermal and epithelial, layers and mediate the interaction between them, this leads to the orderly proliferation and differentiation of these two cell groups and ultimately forms complete hair follicles. The morphogenesis of the hair follicles is a very complex process, which involves an interaction between various types of cells that jointly promote the formation of the hair follicle structure. However, the problems related to the molecular process of hair follicle morphogenesis remain unknown.

The growth pattern of the hair follicles in the yak is similar to that in the mammals such as the mice and cashmere goats [3, 4]. The hair follicles undergo cycles of anagen, catagen, and telogen after periodically growing under the interaction of epidermal and dermal layers [5]. Although the process of hair follicle morphogenesis has been recorded in many kinds of literature, reports that highlight our understanding of the molecular signals and gene regulatory networks in the specific cell groups are limited [6]. Concomitantly, studies on hair follicle morphogenesis have mainly focused on humans and mice such that the studies on the hair follicle morphogenesis of the other species are relatively few.

The anagen is the most active phase of the periodic growth of the hair follicles, during which secondary follicle germs proliferate rapidly in the anagen, making the hair follicles penetrate the subcutaneous tissue. At the same time, the proliferation of the hair bulb cells expand outward, and the hair shaft (HS) and inner root sheath (IRS) continued to differentiate. The induction of anagen largely depends on the interaction of the Wnt, β-catenin, and BMP signaling pathways in the bulge cells [7–9]. Anagen is marked by the upregulation of Wnt, stable expression of β-catenin and inhibition of BMP [10]. But analgen is maintained by important factors such as IGF-1, HGF, VEGF, and the morphogenesis of the hair follicle depends on the Wnt, Shh, Notch, BMP, and other signaling pathways interplay between the epithelial and mesenchymal cells [11]. Recent studies have identified the Wnt, TGF- beta, and Hippo signaling pathways to promote hair follicle growth [12], and p-cadherin to maintain anagen by regulating the canonical Wnt signaling and suppressing TGF-β2 [13].

The hair follicles enter the catagen after anagen. Catagen is a dynamic transition period between the anagen and telogen, which is that mainly characterized by the stoppage in the growth of the hair shaft, and the decrease in the proliferation and differentiation rate of the cells. Degression in the number of cells divided by the hair matrix gradually slows down the differentiation of HS and IRS, and triggers the apoptosis of the hair bulb cells and outer root sheath. The hair shaft stops differentiation and starts separation from places in contact with the dermal papilla. The hair shaft root closes and moves upward to the distal region of the upper part of the hair follicle involved in the periodic change of the hair follicle. The transformation from anagen to catagen mainly involves the interaction between the downstream effect factor of the TNF-α signal and K17, VDR and retinoic acid receptor [14, 15]. TNF-α ensures a timely anagen–catagen transition in pelage follicles and its ablation partially rescues the hair cycling defect of the K17-null mice [16].

Hair follicle cyclic regeneration involves the interaction between the various types of cells and the transmission of the signaling molecules. Classical genetic methods have identified several important signaling molecules in mice for hair follicle cyclic regeneration development. However, there are still many unknown aspects about the molecular regulation of the whole hair follicle development in mice for the non-synchronous development of different types of hair follicles cells. To date, there is a paucity of in-depth studies on the cyclic regeneration of yak hair follicles, and many problems remain unresolved. In recent years, with the rapid development of scRNA-seq, many biological processes that have previously remained elusive have also been described in detail using scRNA-seq technology [17]. This also provides a new means for studying the cyclic regeneration of the hair follicle. Here, a single-cell transcriptome map of the hair follicle was constructed during anagen and catagen. The tSNE analysis identified eight major cell groups and analyzed the molecular characteristics of the different types of cells in detail. The differentiation trajectory of the dermal cell lineages and epidermal cell lineages of the hair follicle during anagen and catagen in yak were constructed based on the pseudotime trajectory. The gene dynamic changes in the process of dermal cell lineage, epidermal cell lineage, HS, and keratinocytes specialization were described in detail. The construction and integration analysis of the single cell transcription map of the yak hair follicle during anagen and catagen, it will provide new insights for better understanding the regulation property of the yak hair follicle cycling.

## Results

### Identification of the major cell types and molecular characteristics of the hair follicle during anagen and catagen

**Fig. 1:**
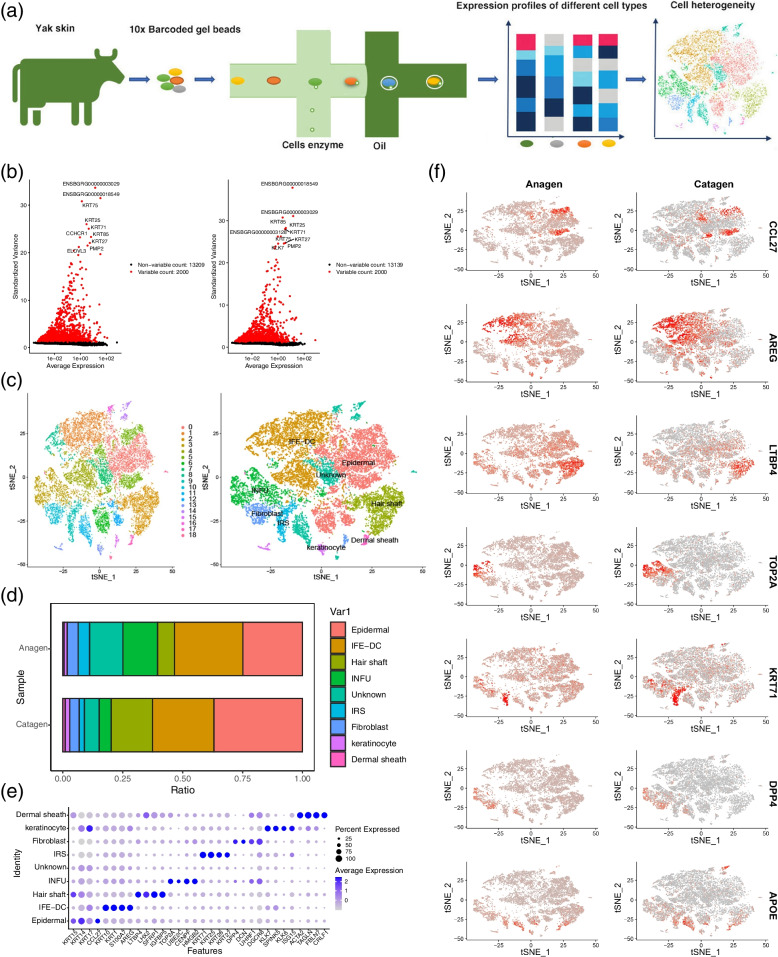
High-variable gene analysis and cell heterogeneity identification of the hair follicle during anagen and catagen. **a** scRNA-seq process; **b** Gene dispersion distribution map; **c** Characterization of the major cell types in the tSNE; **d** Main cell type proportion during anagen and catagen; **e** Comparison of the differential genes in the different cell groups; **f** Expression of the different cell markers in all the cells

The scRNA-seq was used for revealing the cell heterogeneity in the hair follicle during anagen and catagen of the yak (Fig. 1a). To ensure the quality of data, the top 2000 highly variable genes were selected by Seurat for analyzing the subsequent dimensionality reduction and cell clustering. The following figure shows the variation in the gene expression abundance in all the cells during the anagen and catagen obtained by sequencing, and the top 10 genes with the largest coefficient of variation among the cells were selected (Fig. 1b). After quality control and standardization of the scRNA-seq data, the tSNE clustering was analyzed. The cell types were identified for each cluster based on the known marker genes (Fig. 1c), the Clusters 1, 4, and 8 expressed the interfollicular epidermis differentiated cell (IFE-DC) markers *KRT1* [18]*, KRT10* [18]*,* and *SBSN* [18]; the Cluster 2 expressed the HS markers *IGFBP5* [19] and *LHX2* [20]; Cluster 3 expressed the infundibulum (INFU) markers *TOP2A* [18] and *UBE2C* [18]; the Cluster 9 expressed the inner root sheath (IRS) markers *KRT71* [21]*, KRT28* [21]*, KRT27* [21]*,* and *KRT25* [22]; Clusters 10 and 15 expressed the fibroblast markers *DPP4* [23] and *DCN* [6]; the results showed that the clusters 0, 5, 6, 12, and 13 expressed high levels of the epidermal lineage markers *KRT14* [18, 24]*, KRT15* [24]*, KRT17* [24], *SPINK9* [25], and *S100A2* [26], and they were termed as epidermal^*KRT14*+^, epidermal^*KRT15*+^, epidermal^*KRT17*+^, keratinocyte^*SPINK9*+^, and keratinocyte^*S100A2*+^, respectively; Cluster 17 expressed the dermal sheath (DS) markers *ACTA2* [21] and *TAGLN* [21]. It was found that the expression of INFU cells in anagen was significantly higher than that in catagen, and the expression of hair shaft in catagen was higher than that in anagen (Fig. 1d). At the same time, some key genes were selected for each cell type for observing their expression in the different cells (Fig. 1e). The expression levels of *AREG*, *TOP2A*, and *KRT71* in anagen were higher than those in catagen (Fig. 1f).

### Gene enrichment analysis of the different hair follicle cell types

**Fig. 2:**
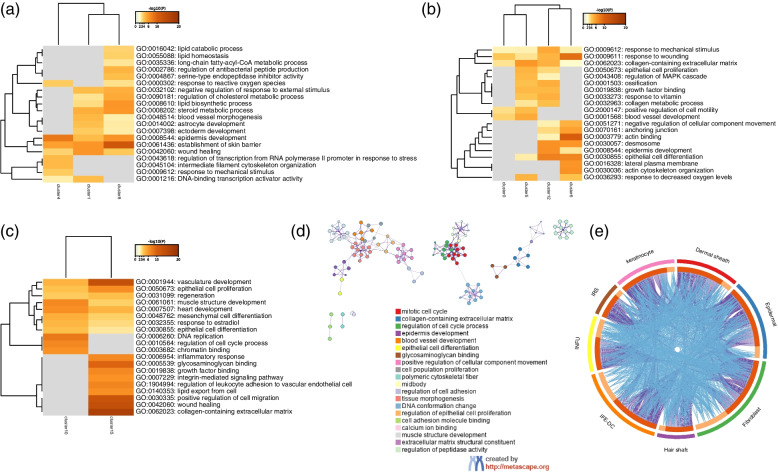
Enrichment analysis of multiple cell clusters. **a** Analysis of the different IFE-DC clusters enriched GO terms; **b** Analysis of the epidermal cell lineage enriched GO terms; **c** Analysis of different the fibroblasts clusters enriched GO terms; **d** GO enrichment network diagram of the different cell types; **e** Gene overlap among the different cell types

To further understand the gene regulation patterns of the different cell types, the GO enrichment was analyzed on the main cell groups after clustering. The IFE-DC (Clusters 1,4 and 8) GO enrichment analysis showed that the three clusters were mainly enriched in wound healing, the establishment of the skin barrier, surface development, and other signaling pathways (Fig. 2a). Epidermal (Clusters 0, 5, 6, and 12) GO enrichment analysis demonstrated these four subgroups were mainly enriched in biological processes such as regulation of MAPK cascade, epithelial cell proliferation, response to wounding, epithelial cell differentiation, and epithelial development (Fig. 2b). The fibroblast (Clusters 10 and 15) GO enrichment analysis showed the two clusters to be mainly enriched in the epithelial cell proliferation, epithelial cell differentiation, muscle structure development, cell cycle regulation, integrin-mediated signaling pathway, wound healing. Cluster15 was enriched in collagen-containing extracellular matrix, wound healing, positive regulation of cell migration, inflammatory response, and epithelial cell proliferation signaling pathways (Fig. 2c). The enrichment analysis of the eight cell types identified these cells to be significantly enriched in the epidermis development, epithelial cell differentiation, regulation of cell cycle process, and other pathways (Fig. 2d). The circos diagram analyzed the overlapping genes between each cell type, the results showed many overlapping genes between these cells (Fig. 2e).

**Fig. 3:**
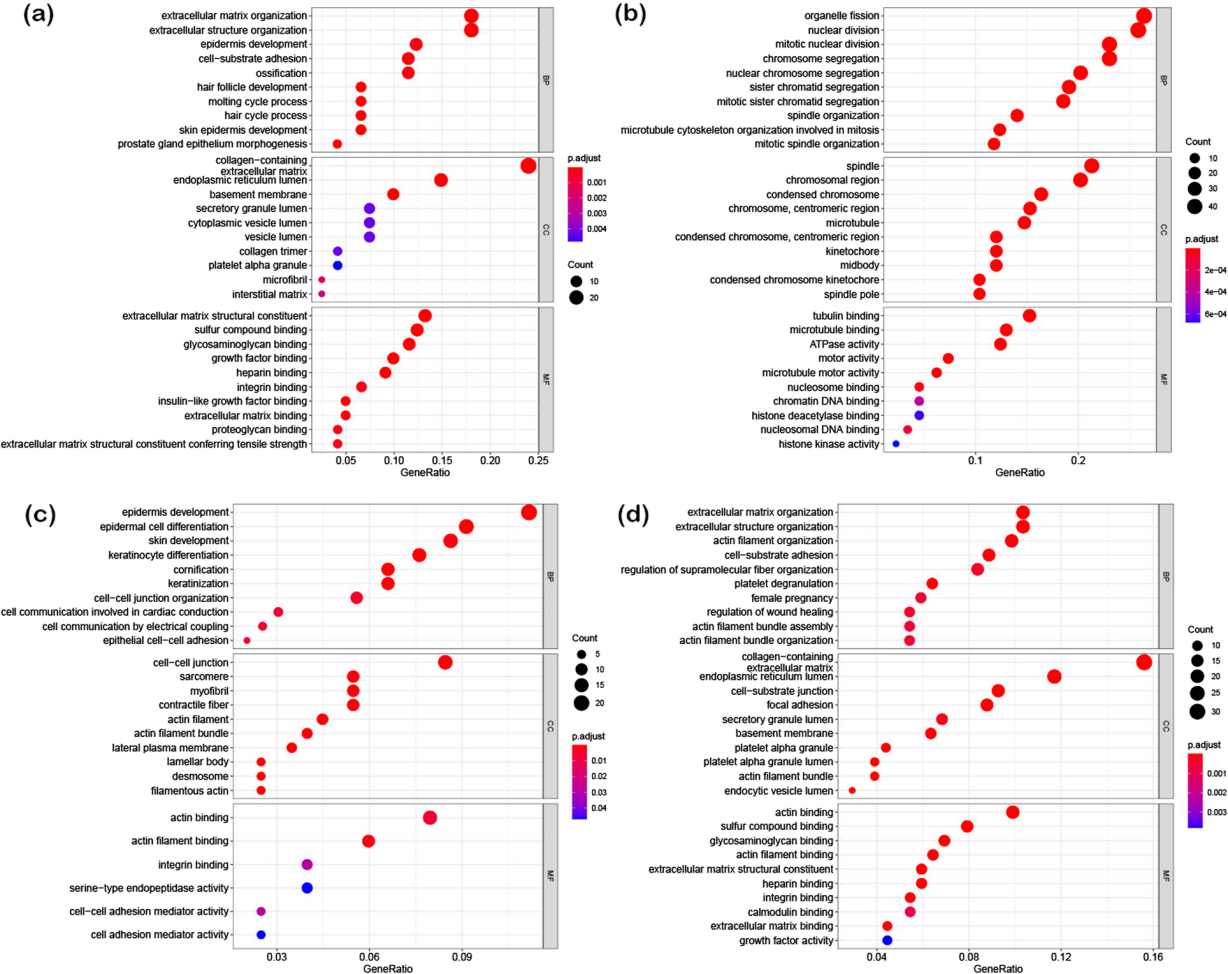
Enrichment analysis of some cell types. **a** The GO enrichment analysis of the HS characteristic genes; **b** GO enrichment analysis of the INFU characteristic genes; **c** GO enrichment analysis of the keratinocyte characteristic genes; **d** GO enrichment analysis of the DS characteristic genes

The differential genes of HS, INFU, keratinocytes, and DS with q-value < 0.05 were screened using the R language, and the top 10 genes of BP, CC, and MF were visualized. The BP of HS was found to be mainly related to the structure of the extracellular matrix, extracellular structure, surface development and cell-substrate adhesion; the CC analysis mainly includes collagen-containing, extracellular matrix, endoplasmic reticulum lumen and other pathways; MF analysis was mainly enriched in the extracellular matrix structural components, sulfur compound binding, glycosaminoglycan binding, growth factor binding and other signaling pathways (Fig. 3a). The analysis of INFU showed BP to be mainly related to the organelle fission, nuclear division, mitotic nuclear division, and chromosome segregation; CC analysis was mainly enriched in the spindle, chromosome region, and condensed chromosome; MF analysis was mainly enriched in tubulin binding, microtubule binding, ATPase activity, and other signaling pathways (Fig. 3b).

The keratinocyte analysis revealed BP to be mainly related to the development of epidermis, epidermal cell differentiation, skin development, keratinocyte differentiation, keratinization, and cell–cell junction structure; The CC analysis was mainly enriched in cell–cell junction, sarcomere, myofibril, contractile fiber, actin filament, and other GO pathways. MF analysis was mainly concentrated in actin binding, actin filament binding, integrin binding, serine-type endopeptidase activity, cell–cell adhesion mediator activity, cell adhesion mediator activity (Fig. 3c). The analysis of dermal sheath showed its BP to be mainly related to the extracellular matrix organization, extracellular structure organization, actin filament organization, cell-adhesion substrate, regulation of fibers organization. The CC analysis was found to be mainly enriched in the collagen-containing, extracellular matrix, endoplasmic reticulum lumen, cell-substrate junction, focal adhesion, and other GO pathways. MF analysis was mainly enriched in actin binding, sulfur compound binding, glycosaminoglycan binding, actin filament binding, extracellular matrix structural component, heparin binding, integrin binding, calmodulin binding, extracellular matrix binding, and growth factor activity (Fig. 3d).

### The pseudotime trajectory analysis of the hair follicle

**Fig. 4:**
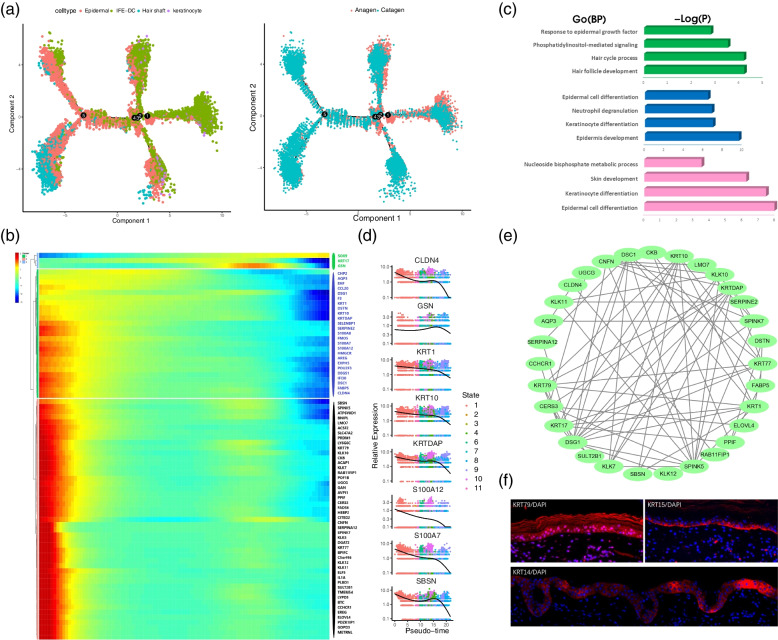
Construction of the pseudotime trajectory of epidermal cell lineage and IFE-DC heterogeneity analysis. **a** Construction of pseudotime trajectories of the epidermal cell lineages during anagen and anagen of yak hair follicles; **b** Gene expression dynamics during IFE-DC specialization;** c** GO analysis of differential genes in the process of IFE-DC specialization; **d** Expression of characteristic genes in different stages of IFE-DC; **e** Interaction analysis of the characteristic genes;** f** Immunofluorescence analysis of skin tissue

The anagen and catagen involve eight types of cells. The pseudotime trajectory analysis of the epidermal cell line obtained from the identification found that the epidermal cell lineage mainly involves the IFE-DC, HS, and keratinocyte developmental trajectory (Fig. 4a). To further explore the gene expression in the developmental trajectory of the different cell types, this study visualized the expression trend of these genes. The monocle analysis was used for studying the differentiation relationship of the epidermal cell lineage. The differential gene expression analysis showed the 1st, 2nd, 3rd, and 4th branches to mainly correspond to the developmental trajectory of the IFE-DC and keratinocytes; the developmental trajectory of HS is mainly concentrated in the fifth branch. The figure demonstrated IFE-DC be highly expressed in the anagen and significantly down-regulated in catagen. The analysis of IFE-DC showed the gene, *SOX9* to be mainly up-regulated during IFE-DC specialization, while *KRT17* and *GSN* were found to be up-regulated first and then down-regulated. The EGF pathway enriched by these genes was found to be closely related to the transformation of hair follicles from the anagen to catagen [27]. The IFE-DC began to expressed *KRT1, KRT10, KRTDAP, SBSN, KLK10,* and other genes (Fig. 4b), mainly enriched in epidermal cell differentiation, skin development, keratinocytes differentiation, neutrophil degranulation, nucleoside bisphosphate metabolic process, and other pathways (Fig. 4c). With time, the biological function of epidermal cell differentiation and keratinocytes differentiation was found to gradually decrease, which also indicated the hair follicles to differentiate from the anagen to catagen. Further analysis of the expression of different genes in different clusters, revealed a downward trend in most IFE-DC marker genes, such as *KRT1, KRT10,* and *KRTDAP* (Fig. 4d). The interaction gene network diagram of the genes encoded in IFE-DC was obtained using the STRING online tool (Fig. 4e). Among these interacting genes, the keratin-related gene *KRT10* was found to be directly correlated with the *KRT1, KRT79, KRT77,* and *KRT17*. In addition, KRT79 and KRT15 were verified by Immunofluorescence. The results showed the expression of KRT79 in the hair follicle epidermal of degenerative yak skin to be significantly higher than that of KRT15, and the expression of KRT14 was also higher than that of KRT15, which was consistent with the results of this study (Fig. 4f).

**Fig. 5:**
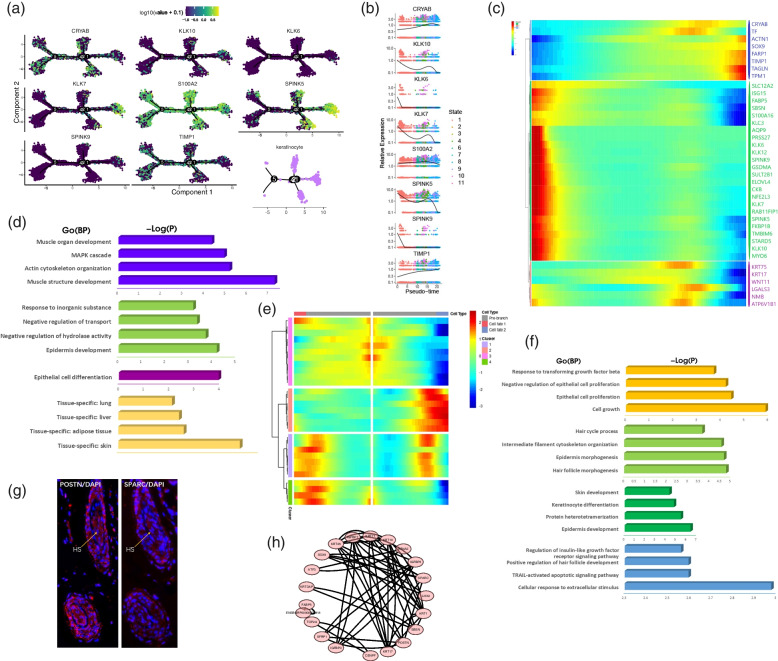
Dynamic changes in the gene expression during the specialization of keratinocyte, HS, and IRS. **a** Expression of the keratinocyte characteristic genes in pseudotime trajectory; **b** Expression of the characteristic genes in different stages of keratinocyte; **c** Gene expression during keratinocyte specialization; **d** Enrichment analysis of differential genes in keratinocyte cell specialization; **e** Gene expression during the specialization of HS and IRS;** f** GO enrichment analysis of the characteristic genes in the process of HS and IRS specialization; **g** Immunofluorescence analysis of the hair follicles; **h** Interaction analysis of characteristic genes in the specialization of HS and IRS

After analyzing the process of specialization of IFE-DC, this study further analyzed the process of specialization of the keratinocytes. The results showed that the pseudotime trajectory of the keratinocytes mainly had three branches. *SPINK5, KLK10, KLK7,* and *SPINK9* were highly expressed in branches 1 and 2, while S100A2 was highly expressed in branches 1, 2, 3, 4, and 5 (Fig. 5a). Analysis of the characteristic genes in the keratinocytes showed that at the beginning of differentiation, the keratinocyte would highly express a series of kallikrein-related genes in specialization, including *KLK6, KLK7, KLK10, KLK12,* and highly express the genes like *SPINK9, CKB, STARD5* (Fig. 5b, 5c). These genes were mainly enriched in the epidermal development, negative regulation of hydrolytic activity, negative regulation of transport, and response to an inorganic substance. Furthermore, the important regulatory roles of these genes in the regulation of epidermal specialization were expounded. The expression levels of *SOX9, FARP1, TIMP1, TAGLN,* and *TPM1* were significantly up-regulated in the keratinocyte specialization stage, while the expression levels of *KRT75, KRT17,* and *WNT11* were up-regulated and then down-regulated during keratinocyte specialization. Interestingly, these genes were found to express not only in the skin but also in the adipose tissues, liver, and lung (Fig. 5d).

This study further analyzed the differential expression of branch 4 and branch 5. The two branches mainly expressed HS and IRS, and HS was found to mainly up-regulate the marker genes *SPARC* and *LHX2* [20]. However, these genes showed a downward trend in IRS (Fig. 5e), and were mainly enriched in cell growth, epithelial cell proliferation, negative regulation of the epithelial cell proliferation, and response to the transforming growth factor β (Fig. 5f). These functions also revealed the growth or maintenance process of the yak hair follicles. TGF-β2 played an important role in inducing the degeneration of the yak hair follicle, which might be mediated by the TGF-βRII to induce epithelial apoptosis [28]. IRS specifically up-regulated the genes *KRT71, KRT25,* and *FABP5,* and were lowly expressed in HS. The Go enrichment analysis indicated these genes to be enriched in hair follicle morphogenesis, intermediate filament cytoskeleton organization and epidermal morphogenesis. For *KRT1, KRT10, KRTDAP,* and other genes which were highly expressed before branching, there were different variation trends in the branches of HS and IRS. These genes were highly expressed at the initial stage of HS specialization and then down-regulated, while they were lowly expressed at the initial stage of IRS specialization and then up-regulated and finally down-regulated. The gene interaction analysis indicated that *KRT10, KRT25, KRT71, KRT17,* and *KRT1* were directly or indirectly linked, while the *IGFBP3, IGFBP5,* and *IGFBP7* of the insulin-like growth factor-binding protein family were closely related (Fig. 5h). IGFBPs are important initiating factors for cell growth. Studies have shown the insulin-like growth factor system to play an important role in hair development and hair follicle cycle control [29, 30]. The above results were verified by immunofluorescence analysis, and the results showed that POSTN and SPARC were all expressed in the HS (Fig. 5g).

**Fig. 6:**
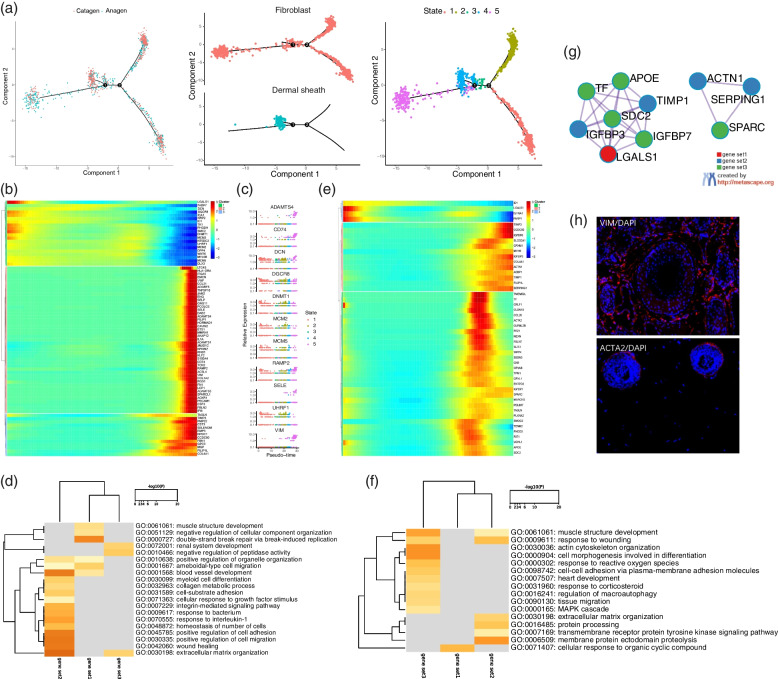
Dynamic changes in the gene expression during specialization of hair follicle fibroblasts and DS. **a** Construction of the pseudotime differentiation trajectory between the fibroblasts and DS; **b** Gene expression during fibroblast specialization; **c** Expression of the characteristic genes in different stages of fibroblast; **d** GO enrichment analysis of fibroblast characteristic genes; **e** Gene expression during DS specialization; **f** GO enrichment analysis of the characteristic genes in DS; **g** Interaction analysis of the characteristic genes in DS; **h** Immunofluorescence analysis of the hair follicles

The pseudotime trajectory inference analysis revealed the first branch to mainly corresponded to the dermal sheath, which was mainly concentrated in catagen. The connective tissue sheath-like cells adjacent to secondary hair buds have been found to participate in the reconstruction of DS during the hair cycle in the anagen I of the hair development, which increases the proliferation of the proximal DS cells, during the hair catagen III, the DS cells begin apoptosis, while during catagen VI, the DS cells disengage from the DS structure and migrate to the nearby dermis [31, 32]. Analyzing the fibroblasts showed them to express in both the branches (Fig. 6a), and highly express in both anagen and catagen, while the DS cells were mainly concentrated in branch 1. During specialization, the fibroblasts were found to specifically up-regulate *VIM, CD74, SELE, ADAMTS4,* and other genes (Fig. 6b, 6c). These up-regulated genes were mainly enriched in extracellular matrix tissues, wound healing, positive regulation of cell migration, and blood vessel development (Fig. 6d). In the initial stage of differentiation, genes such as *DCN, DNMT1, DGCR8,* and *MCM5* were up-regulated, and were mainly involved in the double-strand break repair via break-induced replication, muscle structure development, and negative regulation of the cellular component organization. The genes *ID1, LGALS1, ISYNA1,* and *PARP1* were down-regulated in the DS (Fig. 6e). These down-regulated genes were enriched in the cellular response to the organic cyclic compound. The genes *TIMP3, CCDC80, IGFBP6, IGFBP3,* and *TIMP1* were up-regulated and mainly enriched in membrane protein ectodomain proteolysis, protein processing, extracellular matrix organization and response to wound (Fig. 6f). The expression levels of *ACTA2, TAGLN, CKB,* and *SDC2* were up-regulated in DS and down-regulated at the end of differentiation. These genes are mainly enriched in cell morphogenesis involved in differentiation, tissue migration, cell morphogenesis involved in differentiation, and cell–cell adhesion via the plasma-membrane adhesion molecules. Notably, Previous studies have shown that there may be a differentiation relationship between DS\DP cells and dermal fibroblasts [33]. The interaction analysis of the DS cells gene set was carried out by Metascape, and the results showed *TF, SDC2, LGALS1, IGFBP7, IGFBP3, APOE,* and *TIMP1* to be involved in gene interaction, and *SPARC, ACTN1,* and *SERPING1* to constitute gene interactions (Fig. 6g). In addition, our immunofluorescence results showed that VIM was expressed in fibroblasts and ACTA2 was expressed in DS structure (Fig. 6h).

**Fig. 7:**
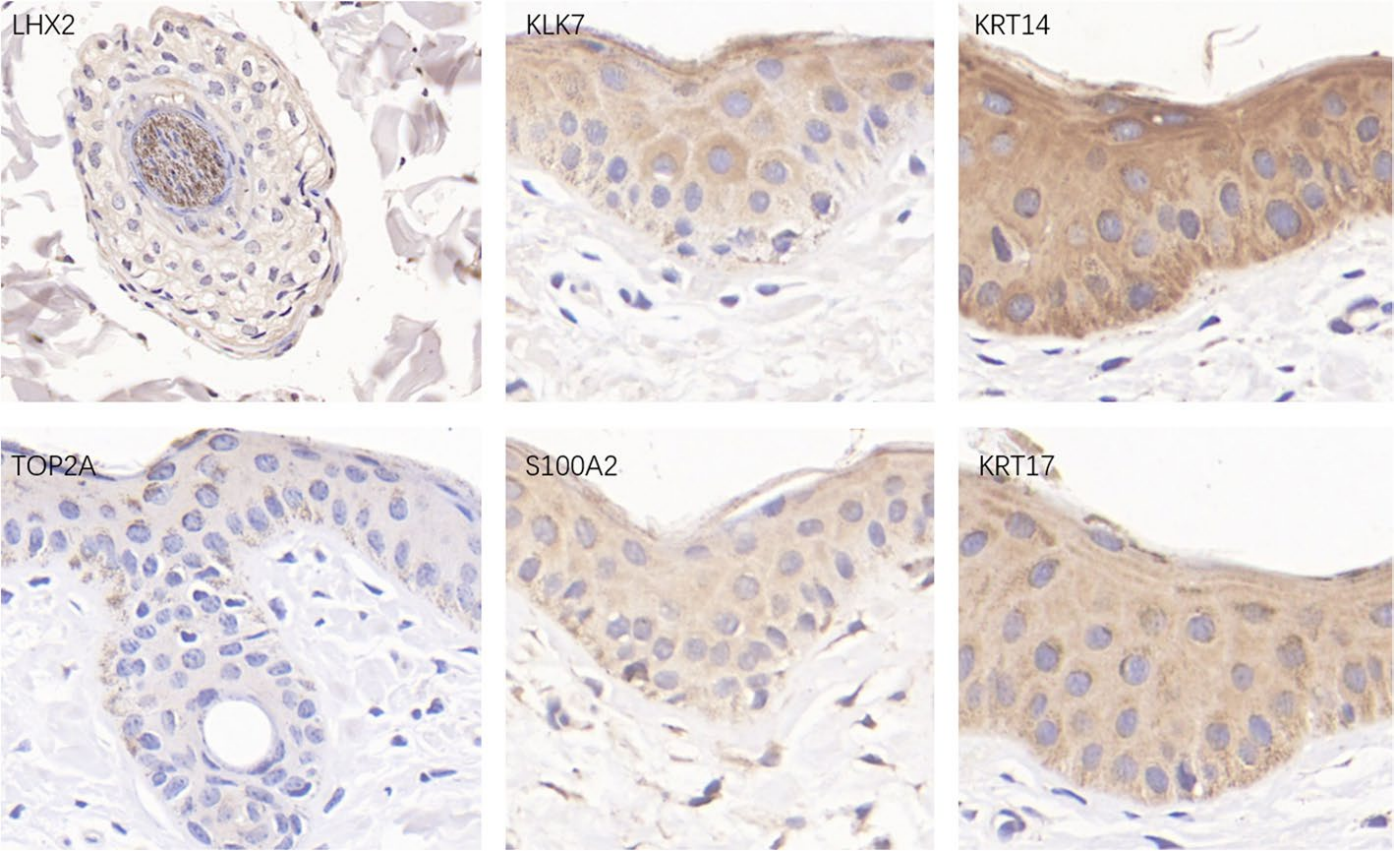
Analysis of the immunohistochemistry of the key protein of yak hair follicles

In order to verify the above analysis results, this study also used immunohistochemistry to perform dyeing analysis of yak skin tissue (Fig. 7). The results show that LHX2 in hair follicles is highly expressed in the hair shaft, and the INFU marker gene TOP2A is low in the hair follicle structure and the epidermal cells around the hair follicles, which is consistent with the above results. In addition, this study also analyzed the expression of the genes of keratinocyte S100A2 and KLK7 in the skin tissue. It was found that the positive expression of S100A2 was mainly positioned in the epidermis tissue between the hair follicles, and the KLK7 protein was high expression. The expression of KRT14 in epidermal was higher than that of KRT17 in the skin tissue of yak hair follicle anagen.

## Discussion

The high-throughput sequencing technology has greatly promoted the in-depth study of the different cell types in complex tissues. The cyclic regeneration of hair follicles is very complex, requiring different types of cell interaction. However, the development of the different types of cells is not synchronized. Studies are still lacking on the mechanisms in model animals. The current studies on yak hair follicle cyclic regeneration are mostly at the stage of morphological description. However, the studies on the hair follicle cycle transformation are limited by the lack of real gene markers in the different cell types, the different reception signals of multiple cells, the crosstalk of different cells, and the change of intermediate cells. This also makes the biological differences in cell interactions possibly covered by the average value, such that it is difficult to accurately describe the specific gene expression profiles of the different types of cells during hair follicle cycling.

With the development of scRNA-seq, many complex biological processes have been described in an unprecedented way. This is particularly useful for cell-level research because the scRNA-seq technology has a powerful ability for analyzing the cell heterogeneity in complex tissues. There are many successful cases of using the scRNA-seq for analyzing the heterogeneity in the morphogenesis of hair follicles [6]. However, there are few reports on the yak hair follicles. Therefore, analyzing the heterogeneity between different types of cells during yak hair follicle cycling using scRNA-seq will be of great significance for revealing the regulatory property of the yak hair follicle periodic growth. The transcriptome profiles of 24,124 single-cells were obtained by scRNA-seq sequencing of the hair follicles in the yak. Based on the reported marker genes and the functional genes of the cell group, each cell subgroup of the hair follicles in the anagen and catagen was identified, and the different cell types involved in developing HF in this period were analyzed. IFE-DC was successfully identified to have three cell clusters, epidermal had four cell clusters, fibroblasts had two cell clusters, while keratinocytes, HS, IRS, DS, and INFU each had one. After analyzing the difference in the expression of genes in each subset, the characteristic genes of the cell cluster-specific expression were identified and enriched by the GO function. The molecular functions and signaling pathways involved in the hair follicles cycle revealed the highly expressed genes in detail.

The cycling of the hair follicle and the transition between anagen and catagen are different cell expression processes. To reveal the property of different cells in the development process, different cells were extracted and analyzed. The epidermal cell lineages are enriched in the regulation of MAPK cascade, epithelial cell proliferation, response to wounding, epithelial cell differentiation, epidermis development, cell-substrate adhesion, and other pathways. The MAPK signal can regulate cell proliferation, differentiation, and apoptosis by affecting gene transcription and regulation. Studies have shown Tβ4 to affect the expression of p-catenin, p38 activation in the MAPK pathway, thereby affecting the growth of the villi, hair follicle distribution, and hair shaft number [34]. The dermal cell lineage was enriched in the epithelial cell proliferation, epithelial cell differentiation, muscle structure development, cell cycle regulation, integrin-mediated signaling pathway, wound healing, cell migration, positive regulation of the epithelial cell proliferation, regulation of supramolecular fiber tissue, and cell–matrix adhesion. These functions contribute to hair cycle regeneration and dermal cell differentiation, thus promoting the integrity of hair follicle structure.

The pseudotime differentiation trajectory analysis described the specific processes of epidermis towards IFE-DC, keratinocyte, HS, and IRS. The characteristic genes *LHX2, KRT25,* and *KRT71* were highly expressed in the specific processes of HS and IRS, and were consistent with the expression results of HS and IRS in the mice [6]. HS and IRS have highly expressed the marker genes of IFE-DC during specialization, and enriched during keratinocytes differentiation and epidermal cell differentiation, indicating that IFE-DC might have direct or indirect links during hair stem and IRS specialization. The pseudotime differentiation trajectory analysis of the differentiation relationship of the dermal cell lineage identified some key regulatory factors involved in the process of DS and fibroblast specialization in the yak hair follicle cycle regeneration that were similar to mice, such as mouse dermal sheath marker molecules *ACTA2* and *TAGLN* [21], and the fibroblast marker molecules *VIM* and *DCN* [6]. The analysis of the second branch of the dermal spectrum identified fibroblasts to be divided into two different specialization directions, with a possibility of unidentified fibroblast subsets. Interestingly, no *SOX2* gene was found while analyzing the whole sequencing data of the yak hair follicles, but was found in the DP cells of the mice and cashmere goats [35], indicating that the yak and other animals differed with respect to the process of hair follicle specialization.

## Conclusions

In summary, this study used scRNA-seq to reveal different cellular transcriptional regulators during anagen and catagen of white yak hair follicles, and the key genes and gene expression profiles of the cell fate during hair follicle cycle transition were described, providing new insights into the direction of cell differentiation and intermediate cell process. At the same time, these results would enrich us by improving our understanding of the cycling of hair follicles.

## Methods

### Experimental animals

All the yaks involved in this study were obtained from the Tianzhu white yak propagation bases of Wuwei City, Gansu Province of China. The experimental procedures were approved by the Animal Care and Use Committees of the Lanzhou Institute of Animal Science and Veterinary Medicine, the Chinese Academy of Agricultural Sciences.

### Analysis of Immunofluorescence and immunohistochemistry

The villi and coarse hairs on the skin surface samples were removed, cut into smaller tissue blocks, and fixed for more than 24 h using a fixative. The target tissue was smoothened, and the dehydration box was put into the dehydrator (Diapath, BG, Italy) with different concentrations of gradient alcohol (Sinopharm Chemical ReagentCo., Ltd, Shanghai, China) for dewaxing and xylene (Sinopharm Chemical ReagentCo., Ltd, Shanghai, China). The tissues were soaked in wax, embedded in the embedding machine, and removed from the dehydration box before the wax solidified. The samples were then cooled at -20 °C, the wax block was removed from the embedding frame and fixed at -20 °C, and sliced on a pathology slicer (Shanghai Leica Instrument Co., Ltd., China), a thickness of 4 μm. The tissue was flattened when the slice floated on the 40 ℃ warm water in the spreading machine, and then, the tissue was affixed to the glass slides and baked in the oven at 60 ℃. The sections were then deparaffinized and and rehydrated, the slides were immersed in EDTA antigen retrieval buffer (pH 8.0) (Servicebio, Wuhan, China) for antigen repair. The excess liquid, was drained off and the objective tissue was marked with a liquid blocker pen (Servicebio, Wuhan, China). The PBS (Servicebio, Wuhan, China) was dried and blocked with 3% BSA (Servicebio, Wuhan, China) for 30 min, and then the diluted primary antibody was added dropwise on the tissue section and incubated at 4 °C in the wet box overnight. The slice was then washed three times with PBS, 5 min each time, and then slightly dried, before adding the secondary antibody (Servicebio, Wuhan, China) dropwise, followed by incubation in the dark for 50 min. After incubation, these tissues were rinsed three times with PBS, 5 min each followed by the addition of spontaneous fluorescence quenching (Servicebio, Wuhan, China) reagent with incubation for 5 min. The slides were then rinsed under flowing water for 10 min and finally mounted with a coverslip with an anti-fade mounting medium. Immunofluorescence using DAPI (Servicebio, Wuhan, China) for nuclear dyeing. Immunohistochemical uses DAB chromogenic reaction, and the sections are counterstained with hematoxylin stain solution. The primary antibodies KRT79, KRT15, POSTN, SPARC, VIM, and ACTA2 were from Bioss (Bioss, Beijing, China). The antibodies LHX2, KLK7, TOP2A, KRT14, KRT17, and S100A2 were from Servicebio (Servicebio, Wuhan, China).

### Preparation of single cell suspension

This study selected three healthy Tianzhu white yaks of about 2 years old as cows. The skin tissue of the scapula was collected during the anagen and catagen of the yak, after cleaning and disinfection, in the tissue protection liquid. The cells were rinsed twice with PBS (Solarbio, Beijing, China) and 75% ethanol in the ultra-clean bench, cut into small strips of 0.5 × 0.2 cm, digested with 0.25% dispase (Sigma, St. Louis, MO, USA) for 2 h in a 37℃ incubator, and a single hair follicle was pulled out, visualized under a stereomicroscope (Olympus, Tokyo, Japan) and placed in a petri dish. After being digested with 0.25% trypsin for 0.5 h, the cells were observed under a microscope to visualize whether they all fell off from the hair shaft. After repeated blows, with 40 μm cell sieve filtration, and centrifugation, the supernatant was aspirated with a Pasteur pipet and resuspended with the medium. Then, the supernatant was centrifuged repeatedly three times and finally, the cells were counted using the cell counter.

### Library preparation and sequencing

The single-cell library was constructed and sequenced by Berry Genomics Corporation (Beijing, China) and the cell was labeled based on the 10xGenomics Chromium™ system. The gel beads containing barcode information were mixed with the cells and beads of oil to form the GEMs. Each gel bead contained a large number of probes composed of Read1, Barcode, UMI, and dT. In each GEM, the mRNA released after the cell rupture was reverse transcribed into the cDNA with Barcode. The cDNA was collected and amplified, and the sequencing library was constructed according to the standard procedure of Illumina sequencing library construction. The size of the InsertDNA in the library was detected using Agilent 2100. After the library was qualified, the PE150 sequencing was performed on the Illumina NovaSeq 6000 platform.

### Sequencing data preprocessing

The original image file obtained by Illumina Novaseq high-throughput sequencing was transformed into Raw Data by base recognition, and then the data were filtered. Removal of adaptor, the number of N > 3, and low quality Reads. The cell detection, reference genome alignment, cell clustering and gene differential expression analysis were performed using the 10 × Genomics official analysis software, CellRanger. The reference genome splicing was identified and the reads were compared using STAR in Cell Ranger. The sequencing errors in the UMI sequence by CellRanger, and the group reads with the same bar code, UMI, and gene annotation were corrected. Only reliable reads could be used for UMI counting. After the CellRanger was completed, the scRNA-seq data were clustered.

### Downstream data analysis of the scRNA-seq

The Seurat package was used for directly reading into the expression matrix after CellRanger analysis, and the Seurat object was created. Then, the low-quality cells were filtered, and the expression percentages of mitochondrial genes, ribosomal genes and hemoglobin genes in the matrix were calculated using the FilterCells function, then use the NormalizeData function to normalize the data. we applied FindIntegrationAnchors and IntegrateData implemented in the Seurat package to remove the batch effects and merge samples into one object. FindIntegrationAnchors took the union of the top 2,000 HVGs from each sample, which was subjected to compute anchors that were finally applied to integrate different datasets. The expression matrix was reduced to important eigenvalues using the dimension reduction algorithm. The principal component analysis (PCA) was used for reducing the dimension of the expression matrix from cells × genes to cell × M. After quality control and scRNA-seq data standardization, the cell clustering results were obtained. At the same time, the signature genes of all the clusters were calculated using the FindAllMarkers function, and the cluster cells were annotated based on the previously reported typical marker genes. Use Monocle for pseudotime trajectory analysis, the dermal cell line cells and epidermal cell line cells were selected as the research objects of Monocle for analysis. The Monocle object is constructed using the newCellDataSet function, and all differentially expressed genes in the cell cluster are screened to construct a minimum spanning tree. Then, the single cell was searched in high-dimensional and low-dimensional space for optimal sorting. Finally, the optimal cell pseudotime trajectory, characteristic gene expression distribution map of each branch and clustering heat map were drawn.

### Enrichment analysis and Interaction analysis

The GO enrichment analysis of differential genes identified the significant relationship between the differential genes in the different groups and biological functions. In this study, the GO enrichment analysis of differentially expressed genes was performed using Metascape (http://metascape.org/). At the same time, the gene name of the regulatory network was transformed into the ID of the gene using the R language. The potential pathways were analyzed using GO enrichment analysis from three aspects: biological process (BP), cellular component (CC), and molecular function (MF). The potential interactions between the coding genes were searched using the STRING database (https://string-db.org/) to construct the interaction network.

## Data Availability

All the sequencing data used in this research is deposited in NCBI GEO databases under accession number: GSE205456.

## Abbreviations

IFE-DC: Interfollicular epidermis differentiated cell
INFU: Infundibulum
IRS: Inner root sheath
DS: Dermal sheath
GO: Gene ontology
MAPK: Mitogen-activated protein kinase
BP: Biological process
CC: Cellular component
MF: Molecular function
