# Correction: Construction of transcriptome atlas of white yak hair follicle during anagen and catagen using single-cell RNA sequencing

**DOI:** 10.1186/s12864-023-09127-5

**Published:** 2023-02-02

**Authors:** Qingbo Zheng, Na Ye, Pengjia Bao, Xiaolan Zhang, Fubin Wang, Lanhua Ma, Min Chu, Xian Guo, Chunnian Liang, Heping Pan, Ping Yan

**Affiliations:** 1 Key Laboratory of Yak Breeding Engineering of Gansu Province, Lanzhou Institute of Husbandry and Pharmaceutical Sciences, Chinese Academy of Agricultural Sciences, Lanzhou, 730050 China; 2 Key Laboratory of Animal Genetics and Breeding On Tibetan Plateau, Ministry of Agriculture and Rural Afairs, Lanzhou Institute of Husbandry and Pharmaceutical Sciences, Chinese Academy of Agricultural Sciences, Lanzhou, 730050 China; 3 Life Science and Engineering College, Northwest Minzu University, Lanzhou, 730030 China

**Correction: BMC Genomics 23, 813 (2022)**

**https://doi.org/10.1186/s12864-022-09003-8**

Following the publication of the original article [1], the authors identified an error in Fig. 6. The correct figure is given below.

**Fig. 6:**
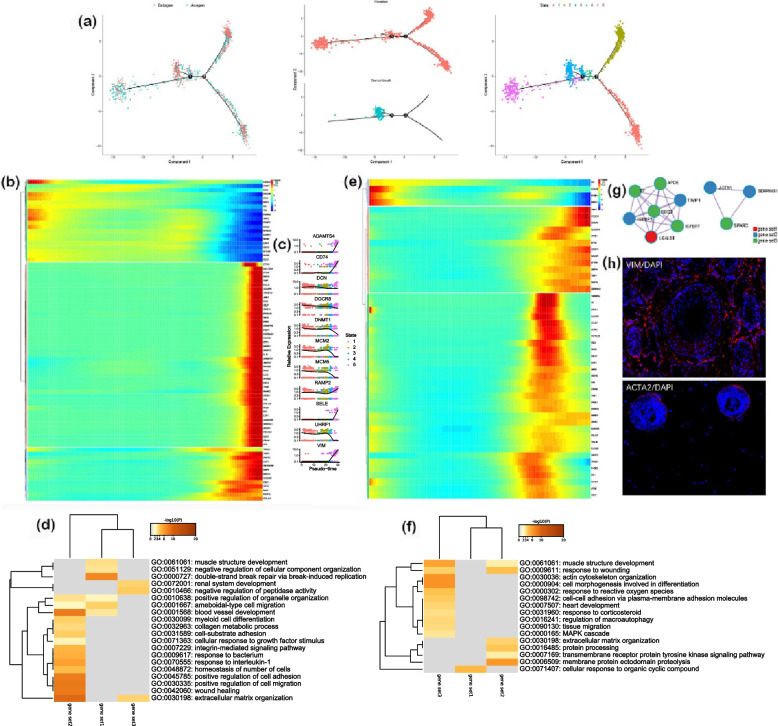
Dynamic changes in the gene expression during specialization of hair follicle fibroblasts and DS. **a** Construction of the pseudotime differentiation trajectory between the fibroblasts and DS; **b** Gene expression during fibroblast specialization; **c** Expression of the characteristic genes in different stages of fibroblast; **d** GO enrichment analysis of fibroblast characteristic genes; **e** Gene expression during DS specialization; **f** GO enrichment analysis of the characteristic genes in DS; **g** Interaction analysis of the characteristic genes in DS; **h** Immunofluorescence analysis of the hair follicles

The original article [1] has been corrected.
